# Chronic Pain in Older Adults: Risk Factors and Prevention

**DOI:** 10.3390/life16091515

**Published:** 2026-09-11

**Authors:** Bruno Daniel Carneiro, Isabel Belo, Sandra Torres, Sofia Tavares Almeida, Isaura Tavares

**Affiliations:** 1 Unit of Experimental Biology, Department of Biomedicine, Faculty of Medicine, University of Porto, 4200-319 Porto, Portugal; bcarneiro@med.up.pt (B.D.C.); up202006294@edu.med.up.pt (I.B.); 2 Rheumatology Service, Unidade Local de Saúde do Alto Minho, Hospital Conde de Bertiandos, 4990-078 Ponte de Lima, Portugal; 3 Unidade de Saúde Familiar São João, Unidade Local de Saúde de Entre Douro e Vouga, 3700-298 São João da Madeira, Portugal; sandra.torres.med@gmail.com; 4 Unidade Local de Saúde de São João, Unidade de Saúde Familiar Valongo, 4440-563 Valongo, Portugal; sofia.tav.almeida10@gmail.com; 5 Institute for Research and Innovation in Health and IBMC (i3S), University of Porto, 4200-135 Porto, Portugal

**Keywords:** chronic pain, older adults, ageing, risk factors, pain prevention, multimorbidity, frailty, lifestyle factors, early intervention, pain modulation

## Abstract

Chronic pain is highly prevalent among older adults and represents a major health challenge due to its multifactorial aetiology, association with multimorbidity and frailty, and impact on quality of life. This narrative review synthesises evidence from the last few decades addressing chronic pain in older adults, with emphasis on risk factors, underlying mechanisms, and preventive strategies. Risk factors were organised according to the Dahlgren and Whitehead “Rainbow model” into individual, environmental, and social determinants. Individual-level factors—particularly physical inactivity, abnormal body weight, poor nutrition, mental health disorders, maladaptive pain beliefs, sleep disturbance, previous surgical and postoperative pain, multimorbidity, frailty, smoking, and alcohol use—were associated with pain persistence. Emerging evidence related to environmental and social determinants, including reduced nature and sunlight exposure, low socio-economic status, occupational factors, and social isolation, suggests further increased pain vulnerability. Mechanistic evidence indicates that ageing is associated with impaired descending pain modulation and increased susceptibility to peripheral and central sensitisation. Although randomised preventive trials in older adults remain scarce, converging evidence suggests that targeted lifestyle modification, early pain management, and multimodal non-pharmacological strategies may reduce progression to chronic pain. A prevention-oriented, risk-stratified approach integrating clinical care with broader public health strategies may help mitigate the growing burden of chronic pain in ageing societies.

## 1. Introduction

The International Association for the Study of Pain (IASP) and the International Classification of Diseases, 11th Revision (ICD-11) define chronic pain as pain that persists or recurs for longer than 3 months [1]. The distinction between chronic primary and chronic secondary pain [1] is also relevant to prevention, because in chronic secondary pain, preventive strategies may in part target the underlying disease, injury, or treatment-related factors contributing to persistent pain, whereas chronic primary pain may require greater emphasis on modifiable biopsychosocial factors and early prevention of pain-related disability. This distinction is considered a conceptual framework rather than as the principal organisational structure of this review. Older adults often exhibit multifactorial chronic pain (nociceptive, neuropathic and nociplastic) [2,3] due to degenerative musculoskeletal conditions (e.g., osteoarthritis, degenerative spine disease), neuropathies (e.g., chemotherapy-induced neuropathy, diabetic peripheral neuropathy, post-herpetic neuralgia), vascular insufficiency, frailty, sarcopenia and low-grade systemic inflammation. Ageing is also associated with changes in the neurobiological mechanisms of endogenous pain modulation, namely in the brain [4,5].

Chronic pain is one of the most common and debilitating conditions affecting older adults. As the global population ages, the burden of chronic pain in older adults is becoming increasingly significant for individuals and healthcare systems. The worldwide prevalence of chronic pain is estimated to be between 25% and 50% in older adults living in the community and up to 83% in institutionalised individuals [6,7]. In Portugal, chronic pain prevalence is over 55% at ages between 65 and 69 years and over 62% at ages over 75 years [8]. In Sweden, a longitudinal study found a prevalence of 38.5% among adults aged 65 to 103 years [9]. A Chinese community-dwelling study reported a prevalence of 57% among older adults aged 60 years [10]. In Brazil, a systematic review estimated the prevalence of chronic musculoskeletal disorders reaching 86% in older adult populations [11]. In a nationally representative United States of America (USA) sample of adults aged ≥65 years, a chronic pain prevalence of 15.9% was reported using a definition of self-reported pain lasting for ≥3 months in the preceding year [12]. The substantial variation in reported prevalence may reflect differences in study populations and settings, but also differences in case definitions, diagnostic criteria, assessment instruments, and methodological approaches used to identify chronic pain. Body areas with more common chronic pain complaints in older adults are the lower back (reported by approximately 50–55% of older adults with chronic pain), knees, legs/feet, neck, shoulders, and major joints [13,14,15]. Musculoskeletal pain, particularly in the lower back and knees, is consistently reported as the most prevalent, followed by pain in the upper back, neck, and shoulders [13,14,15,16]. Chronic pain in the legs and feet is also highly prevalent, especially in community-dwelling older adults [16]. Pain is frequently multisite, with up to 43% of older adults with chronic musculoskeletal pain reporting pain in four or more locations [15]. As populations age globally, and as survival with chronic diseases improves, the number of older adults living with chronic pain is increasing [17]. Healthcare systems and social care services will face growing demands for pain management in older populations. Older adults with chronic pain typically have higher healthcare use, polypharmacy, hospitalisations, and overall cost burden (though precise data by older-adult subset are less often broken out) [3,18,19].

In addition to being highly prevalent, chronic pain has been linked to several detrimental impacts in older people, including mood disturbance, functional and cognitive impairment, poorer sleep and reduced quality of life [20]. As for the latter, pain limits mobility, daily activities and instrumental activities of daily living (IADL) [21]. For example, in a Brazilian study, approximately 54.6% of older adults reported high-intensity chronic pain in locations that compromise IADL [22]. A longitudinal study (Survey of Health, Ageing and Retirement in Europe) found that moderate or severe pain and multisite pain were associated with increased risk of falls (odds ratio of 1.35 or 1.52, respectively) among adults aged 50 years or more over two years [23]. Chronic pain also correlates strongly with frailty state: one prospective analysis of three cohorts found frail individuals had a minimum of 10 and a maximum of 13 plus higher odds of pain compared to robust individuals [24]. Chronic pain is associated with depression, anxiety, insomnia and poorer self-perceived health [25,26,27,28]. Some evidence suggests associations between chronic pain and cognitive decline or mortality, though causality remains unclear [29]. However, one 24-year longitudinal study found no significant multivariate association between chronic pain and risk of dementia in older adults (adjusted hazard ratio of 1.23) [30]. A recent cohort found that among older adults (more than 60 years), social isolation or loneliness were significant risk factors for chronic pain onset (odds ratio of 1.21 for social isolation and odds ratio of 1.61 for loneliness) over seven years [31]. In summary, studies consistently demonstrate that every measured dimension of health is worse when chronic pain is present than when it is absent. It is also known that, in people with other medical comorbidities, chronic pain may be an independent risk factor for all-cause mortality [32]. Given all the abovementioned data, chronic pain in older adults is not just a symptom but a complex, multidimensional condition with major personal, functional and societal consequences that deserves increased attention from the scientific and medical communities.

The management of chronic pain in older adults follows standard principles and should be multimodal, multidisciplinary and personalised, but requires adaptation for older-age physiology, comorbidity and polypharmacy [3,18,33,34,35,36]. A global geriatric assessment to evaluate all the health issues is also relevant in the context of chronic pain. Non-pharmacological treatments (exercise, physical therapy, behavioural therapies, sleep hygiene) are relevant strategies and often preferred before escalating pharmacologic therapy in frail older adults [3,18,33,34,35,36]. Ageing leads to reduced renal and hepatic clearance, altered volume of distribution, changes in receptor sensitivity, decreased homeostatic reserves and increased sensitivity to sedatives/opioids [37,38]. Polypharmacy is highly prevalent among older adults (30 to 50% in some studies) and increases risk of adverse drug reactions, duplications, cumulative toxicity and falls [24,35]. In the context of chronic pain, additional analgesics, adjuvants (e.g., gabapentinoids, tricyclic antidepressants, serotonin–norepinephrine reuptake inhibitors), opioids and topical agents may further increase risk. Also, older adults are at greater risk of nonsteroidal anti-inflammatory drug-related adverse events and benzodiazepine interactions when used concomitantly. While polypharmacy is often necessary to manage multiple chronic conditions in older adults, it can exacerbate chronic pain through adverse drug reactions, drug–drug interactions, and iatrogenic harm, underscoring the need for careful medication review and deprescribing strategies [35,39,40]. This complex issue frequently leads to a clinical and ethical problem: older adults are sometimes under-treated because of fear of side effects.

The response to pain treatments in older adults may be blunted by comorbidities (e.g., cognitive impairment, frailty, sleep disturbance), psychosocial factors, and central sensitisation, meaning that standard treatments may yield less benefit. Structured exercise (strength, balance, aerobic) reduces pain intensity, improves function and reduces risk of disability in older adults with musculoskeletal pain [3,18,36]. Cognitive behavioural therapy, mindfulness-based interventions, and commitment therapy help with pain coping, reduce catastrophising and improve psychological resilience [41,42,43]. Since sleep disturbances interact with pain, treating insomnia or poor sleep (via behavioural sleep medicine and sleep hygiene) is critical [28,44]. Weight reduction in obese older adults with osteoarthritis improves pain and function [45,46,47]. Where appropriate, nerve blocks, neuromodulation or minimally invasive spine interventions may be considered, but with caution in older adults (due to comorbidity, anaesthetic risk, recovery capacity) [48,49,50].

Given the high prevalence of chronic pain, complex causes, and the limitations of current treatments [51], it is important to understand potential risk factors, especially modifiable ones, to try to prevent the onset of chronic pain. We do not specifically address the gender issue in our review, as it was discussed in detail recently [52], with indications that elderly women are a population that should be under special surveillance. In this narrative review, we explored the putative risk factors and the role of prevention in the chronic pain of older adults, allowing a better understanding of this public health problem and targeting new principles of the management of this problem and new directions of research.

## 2. Materials and Methods

This study was conducted as a narrative review of chronic pain in older adults, with emphasis on risk factors, mechanisms relevant to pain persistence, and prevention-oriented approaches. The review was informed by literature identified through searches of MEDLINE/PubMed and Scopus.

For the purposes of this review, older adults were defined primarily as individuals aged ≥65 years. However, because age thresholds varied across the literature, studies using lower or higher cut-offs were not automatically excluded; their differing population definitions were considered when interpreting the evidence.

The databases were searched in November 2025 and updated in April 2026. The search strategy combined terms related to chronic pain, older adults, risk factors, prevention, ageing, and relevant individual, environmental, and social determinants using Boolean operators. The core search concepts included (“chronic pain”) AND (“older adults” OR elderly OR ageing) AND (“risk factors” OR predictors OR prevention), with additional targeted searches performed for specific domains discussed in the review, including physical activity, body weight and nutrition, mental health, sleep, previous pain, surgery, comorbidity, frailty, environmental exposures, socio-economic factors, occupational factors, social isolation, and loneliness.

Studies published in English and addressing older adults were prioritised. Because this was a narrative rather than a systematic review, no formal study-level screening procedure or quantitative synthesis was conducted. Articles were selected by the authors based on relevance to the objectives of the review, clinical and methodological relevance, and their contribution to the conceptual framework of risk stratification and prevention. Reference lists of relevant articles were also examined to identify additional pertinent evidence. Priority was given to evidence derived from older-adult populations. Where evidence was obtained from mixed-age or general adult populations, this was explicitly considered when interpreting its applicability to older adults.

The review considered literature published primarily over the preceding three decades, while earlier studies were retained when considered seminal or necessary to provide historical or conceptual context. Evidence was critically and qualitatively synthesised by the authors. In the review, we used the Scale for the Assessment of Narrative Review Articles (SANRA) [53] when applicable, which can be consulted in the Supplementary Material (Table S1).

## 3. Risk Factors for Developing Chronic Pain in Ageing and Modifiable Targets

There is evidence for some risk factors which may predispose older adults to the development or persistence of chronic pain, and which therefore may serve as prevention targets. We present the results organised according to the well-known “Rainbow model” of the determinants of health initially proposed by Dahlgren and Whitehead in 1991 [54]. According to this model, there are 3 major groups of risk factors: individual, environmental and social.

**Figure 1:**
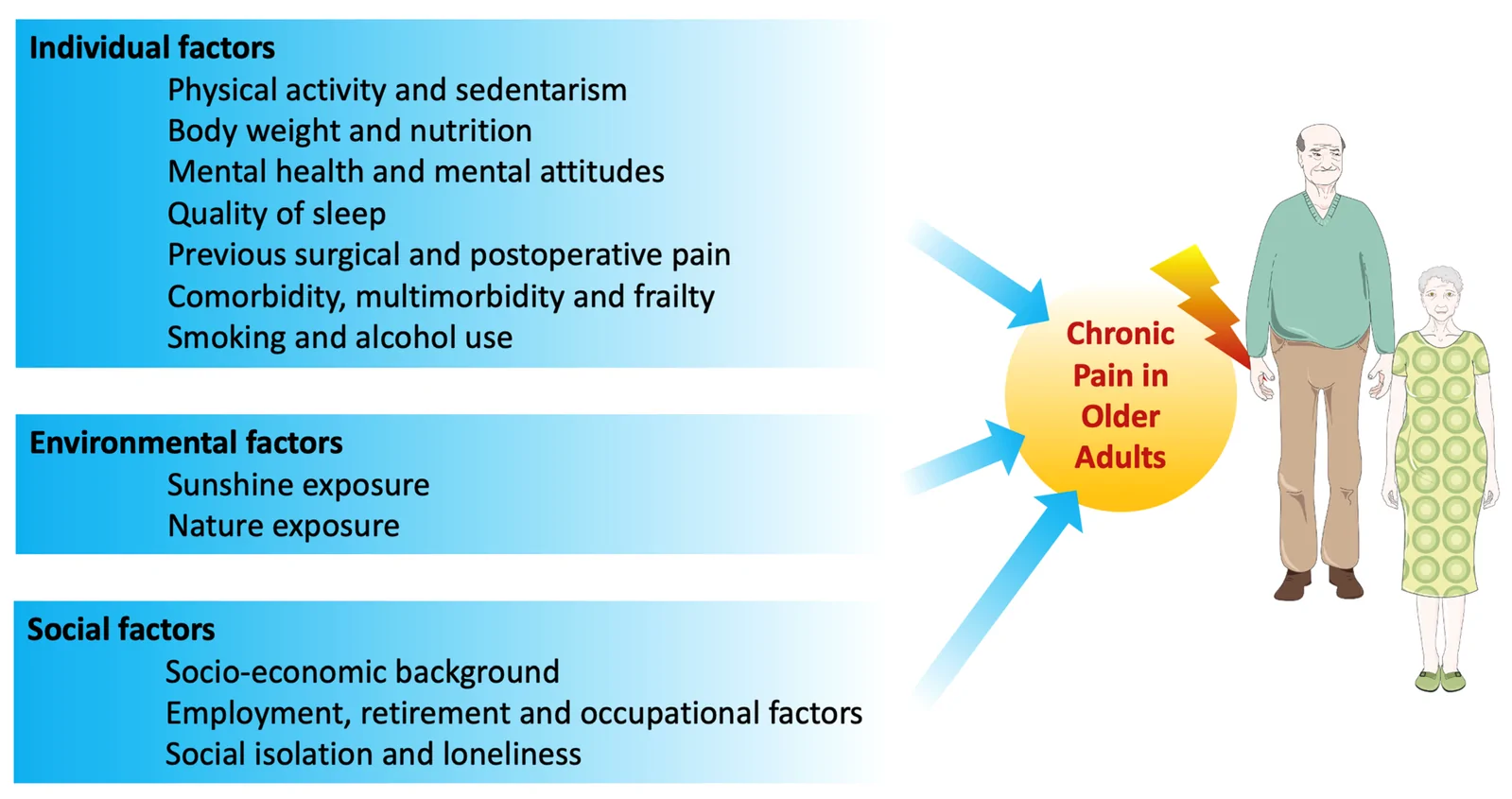
Risk factors for developing chronic pain in the elderly according to the “Rainbow model”. Parts of this figure were drawn using pictures from Servier Medical Art by Servier (https://smart.servier.com), which is licensed under Attribution 4.0 International (Creative Commons CC BY 4.0), and finalised utilising Microsoft PowerPoint software version 16.112.1 (Microsoft Corporation, Redmond, WA, USA).

A summary of the risk factors discussed below is shown in Figure 1 and Table 1.

### 3.1. Individual Factors

According to the results of the analysis of the literature, the main risk factors to develop chronic pain in late life are related to physical conditions and health habits of each individual.

Importantly, older adults should not be considered a homogeneous population. The relevance and feasibility of preventive strategies may vary substantially across the ageing spectrum, particularly between younger-old, old-old, and oldest-old adults. In very old or frail individuals, preservation of functional capacity, muscle mass, nutritional status, and independence have priority over interventions such as intentional weight loss. Similarly, lifestyle modification may be more difficult to implement in the oldest age groups because of frailty, multimorbidity, functional limitations, or cognitive impairment. Psychological interventions such as cognitive behavioural therapy may also require adaptation according to cognitive and functional status. Therefore, pain prevention should be individualised according to biological age, frailty, comorbidity, cognitive status, and functional capacity rather than chronological age alone.

#### 3.1.1. Physical Activity

Evidence shows that physical inactivity or low levels of exercise are associated with higher risk of incident pain and poorer outcomes once pain has developed [55,56]. Interestingly, a longitudinal study of older men found that among those without low back pain at baseline, 28% reported pain at 24 months and that persistent pain at 24 months among those with pain at baseline was 58% [57]. Similarly, a longitudinal “lifestyle aspects” cohort of oldest-old (75 years old or older) individuals found that lifestyle behaviours are associated with pain risk [58]. Evidence from review literature suggests that physical activity and exercise may be associated with a lower risk or burden of chronic pain in older adults [59,60]. Exercise is likely to be important in prevention and management of comorbidities [61].

#### 3.1.2. Body Weight and Nutrition

Evidence specifically addressing the relationship between body weight and chronic pain in older adults is more limited than that available in general adult populations.

Overweight and underweight correlate with higher pain prevalence in older adults, likely via sarcopenia, frailty, systemic inflammation and mechanical load [12,62,63], as referred to in the next section. Obesity, defined as a body mass index (BMI) greater than 30 Kg/m^2^, is related to multimorbidity and is associated with chronic pain. Obesity increases chronic pain in several ways, including placing strain on weight-bearing joints, reducing physical activity, and contributing to overall body deconditioning. A large-scale population study found that the likelihood of reporting chronic pain increased proportionately with BMI [62]. This increased prevalence of chronic pain is seen even after adjusting for the impact of obesity on other medical conditions, which contribute to multimorbidity and which are independently associated with pain [32,64,65]. The relationship between obesity and chronic pain is more complex than simply one of mechanical overload, as demonstrated in a community-based twin study [66], where familial (genetic and environmental) factors were significant contributors to the association. In obese older adults, there seems to be an increased prevalence of chronic pain among those with central obesity that is independent of other components of the metabolic syndrome, depression, anxiety and the presence of painful comorbid conditions. In these older adults, a higher BMI was clearly associated with an increased incidence and prevalence of pain [61,64,66]. Regarding underweight and frailty, the latter itself is strongly associated with chronic pain. One multi-cohort study found a pre-frail odds ratio of 3.8 (minimum) and 4.3 (maximum) and frail odds ratio of 10 (minimum) and 13 (maximum) for pain compared to robust adults [24].

In older adults, malnutrition and micronutrient deficiencies may be particularly relevant because of their contribution to frailty, sarcopenia, and impaired recovery. However, the role of nutrition in the development and prevention of chronic pain remains to be established. It is well-established that malnutrition, micronutrient deficiencies and sarcopenia promote frailty and may contribute to pain via poorer musculoskeletal function and recovery capacity [67,68,69]. Nutrition management plans may be of benefit to patients with chronic pain by improving pain management and reducing cardiovascular risk factors that are related to chronic pain [32,70]. A relationship between high levels of reported pain and low levels of vitamin D has been demonstrated, with the suggestion that low vitamin D levels cause anatomic, endocrine, neurological, and immunological changes, which predispose individuals to onset and perpetuation of chronic pain. However, the effect is not replicated across all studies. However, this association has not been consistently demonstrated across studies, and the clinical significance of vitamin D status for chronic pain remains uncertain [32,71,72,73]. Recent studies show that gut microbiota-derived mediators can regulate peripheral sensitisation of pain, either positively or negatively. For instance, bile acids (BA) can inhibit pain, inducing opioid release from macrophages and decreasing the excitability of dorsal root ganglion neurons. Alternatively, BA can activate the farnesoid X receptors (FXR), prompting mast cells to release the neurotrophic agent nerve growth factor (NGF), and augment the pain intensity [6,74]. These findings were derived from experimental studies in rats and mice, and therefore their relevance to chronic pain in older adults remains uncertain. The role of nutrition in pain development in older adults is an area in expansion.

#### 3.1.3. Mental Health and Mental Attitudes

Older adults with psychopathological conditions such as depressive symptoms and anxiety are at higher risk of chronic pain development [75,76,77,78]. An older-workers cohort found that high stress (odds ratio of 1.62) is associated with chronic lower back pain [75]. Anxiety, depression and catastrophising beliefs about pain have been shown to be associated with the presence of chronic pain and with a poor prognosis in people with various pain conditions. The temporal relationship between chronic pain and mental health remains unclear, and it is likely that there is a “bi-directional aetiology”: pain causing poor mental health and vice versa. In depressed patients, neuroimaging provided evidence of disturbed prefrontal brain activity and a dysfunction of emotion regulation during experimental pain stimulation. This reinforces how factors, such as depression and anxiety, associated with chronic pain become part of the overall condition itself and augment the pain experience [61,76,79]. Anxiety and fear about pain are linked to a higher likelihood of developing chronic pain and a poorer prognosis of recovery from chronic pain. Fear avoidance behaviours and associated lack of movement are independent risk factors for developing chronic pain [32,77,78].

Among older adults, emotional and cognitive factors may influence both pain perception and the risk of persistent pain. Depression, anxiety, pain-related fear, and catastrophising may affect nociceptive processing and descending pain modulation, while age-related changes in attention and cognitive function may further influence pain perception [61,76,79,80].

It is well documented that attitudes and beliefs about pain may affect chronic pain in older adults. Personal beliefs and attitudes can affect a person’s likelihood of developing long-term pain or pain-related disability. Studies have linked passive coping strategies, such as resting and relying primarily on medication, with greater healthcare use and pain-related disability [32,81]. In older adults, such maladaptive coping strategies may be particularly relevant because they may further contribute to physical inactivity and functional decline.

#### 3.1.4. Quality of Sleep

Sleep disturbance is associated with chronic pain. Poor sleep and insomnia are independent risk factors of incident pain and of worse pain outcomes [75]. An older-workers cohort found that poor sleep habits (odds ratio of 1.66) are associated with chronic lower back pain [75]. Additionally, older adults frequently suffer sleep fragmentation, which may increase central sensitisation to pain [82].

The relationship between sleep and chronic pain in older adults is multifactorial and is influenced by age-related changes and comorbid conditions. Obstructive sleep apnea, obesity, chronic obstructive pulmonary disease, cardiovascular disease, and stroke may independently contribute to sleep disruption and daytime symptoms, while chronic pain may further impair sleep quality. Thus, poor sleep should not be considered solely a consequence of pain, but rather as part of a complex interaction between pain, comorbidity, and age-related vulnerability.

A prospective survey from Norway, over a 17-year period, found that disrupted sleep was a risk factor for the onset of chronic pain and predictive of pain persistence (but not worsening of pain) [61,83].

The association is bidirectional and multifactorial, with chronic pain potentially disrupting sleep while sleep disturbance may increase pain intensity and persistence; comorbidities and age-related changes may further contribute to both outcomes. Sleep deprivation was found to be a risk factor for chronic pain in the prospective survey referred to above [32,83,84], but a direct relation should be better studied.

In addition to insufficient or fragmented sleep, excessive daytime sleepiness and unusually long sleep duration may also be relevant markers of poor health and vulnerability to chronic pain in older adults, although their independent predictive value requires further investigation.

#### 3.1.5. Previous Surgical and Postoperative Pain

One of the most important risk factors for the development of chronic pain is the presence of acute or chronic pain within the body. The greater the severity and the greater the number of sites, the more likely chronic pain is to develop. The presence of painful stimuli alters brain neurochemistry in such a way as to predispose individuals to develop chronic pain, probably due to sensitisation of the nervous system. This increased susceptibility to pain can develop within days of exposure to continuous painful stimuli and can persist for up to a year after the pain has been resolved. Effective treatment of acute pain may reduce persistent nociceptive input and could potentially reduce the risk of chronicity, although direct preventive evidence remains limited [85,86,87]. One of the most important ways to reduce the incidence of chronic pain is to prevent acute pain from occurring and manage it well when it does occur [32,87].

Chronic post-surgical pain (CPSP) is not unique to older adults, and its underlying mechanisms are broadly shared across age groups, involving persistent nociceptive input, inflammation, peripheral and central sensitisation, nerve injury, and altered pain modulation. However, older adults may have distinctive risk profiles because of age-related changes in pain processing, multimorbidity, frailty, pre-existing pain, polypharmacy, and reduced physiological reserve. These factors may increase vulnerability to persistent post-surgical pain and may also complicate its recognition, assessment, and management. Therefore, the risk of CPSP in older adults should be considered within the broader context of preoperative pain burden, surgical factors, postoperative complications, and individual biological and functional vulnerability.

There is some evidence to suggest that brain changes associated with chronic pain may be reversible after effective treatment. A recent neuroimaging study in healthy individuals found that brain plasticity can be induced by repetitive experimental noxious stimuli as early as 8 days (after daily pain stimulus for 8 consecutive days), and that this receded between 22 days and 12 months later after the pain stimuli ceased. These neuroplastic brain changes occurred in the early stages of pain (before pain could be labelled as chronic), further suggesting that early intervention will be important in preventing chronicity, though this remains to be tested clinically [61,87,88].

In the abovementioned settings, iatrogenic pain needs to be considered, namely surgical and postoperative pain. Postoperative chronic pain is a significant complication of many surgical procedures. CPSP incidence varies substantially according to the surgical procedure, definition of chronic pain, and patient characteristics. While relatively low rates have been reported in some surgical populations, substantially higher rates have been described after specific procedures, particularly those involving nerve injury or extensive tissue trauma. It is particularly common after amputations (50–85%), thoracotomies (5–65%), cardiac surgery (30–55%), and breast surgery (20–50%). Patients who were anxious about their operation and those who developed postoperative infections were also more likely to suffer from CPSP [32,89,90]. The mechanisms of CPSP are complex and ill-understood. Different mechanisms will be responsible for different pain syndromes even after the same operation, for example, phantom pain, stump pain, and back pain after lower limb amputation. Clearly, many of the syndromes are neuropathic and result from changes in the nervous system after injury. Surgery should be seen as an injury, obviously in most cases necessary and performed for good reason, but nonetheless an injury. An understanding of the types of changes that occur in the nervous system after disease and injury is important, but the subject is complex and needs further investigation [89]. Interestingly, chronological age does not appear to consistently increase the risk of chronic post-surgical pain. Indeed, several studies have reported lower rates of chronic post-surgical pain among older patients compared with younger adults [91,92]. This apparent inverse association highlights the heterogeneity of chronic pain mechanisms across clinical contexts and cautions against assuming that older age uniformly increases susceptibility to persistent pain after surgery.

Frailty, cognitive impairment, multimorbidity, polypharmacy, and reduced physiological reserve may further complicate perioperative pain assessment and recovery.

#### 3.1.6. Comorbidity, Multimorbidity and Frailty

Multiple studies demonstrate that older adults with more comorbid conditions (e.g., osteoarthritis, neuropathy, chronic obstructive pulmonary disease, sleep apnoea) are at higher risk of chronic pain [93,94]. Patients with comorbid physical and mental chronic diseases are more liable to suffer chronic pain than those without [32,94].

A retrospective outpatient review with 2431 patients who were more than 65 years found that patients with chronic pain had a significantly higher prevalence of depression (22% vs. 15%), anxiety (27% vs. 17%), chronic obstructive pulmonary disease (9% vs. 6%), obstructive sleep apnoea (17% vs. 12%), osteoarthritis (49% vs. 26%), and peripheral neuropathy (14% vs. 5%) [93]. A cross-sectional study found that unhealthy body weight (underweight and obese) was associated with increased chronic pain in older adults [12]. Frailty itself is important: one multi-cohort study found a pre-frail odds ratio of 3.8 (minimum) to 4.3 (maximum) and frail odds ratio of 10 (minimum) to 13 (maximum) for pain compared to robust older adults [24].

The prevalence of chronic pain is higher in those with other chronic diseases than in those without. There is evidence that several chronic physical conditions may increase the risk of chronic pain. This may occur directly, through increased nociception from the periphery, resulting in central and peripheral pathophysiological changes associated with chronic pain, or indirectly, by accumulated stress or load, with prolonged activation of the stress-regulation systems leading to breakdown of muscle, bone and nerves, resulting in more pain. This means that other comorbid conditions (not associated with chronic pain) are likely to contribute to the reporting of chronic pain [61,94]. Long-lasting unresolving disorders, such as dementia, produce ongoing nociceptive or neuropathic stimuli and might be entirely responsible for chronic pain in older adults [6].

#### 3.1.7. Smoking and Alcohol Use

While older-adult-specific longitudinal data are relatively limited, alcohol and tobacco remain modifiable behavioural risk factors. In older men, alcohol increased persistent pain odds [57]. Evidence regarding alcohol consumption and chronic pain in older adults is limited. In the study cited, the association was observed specifically among men, and therefore these findings should not be generalised to older women. Tobacco impairs microvascular circulation, increases systemic inflammation, impairs healing and is associated with many pain conditions [95,96]. Smoking has been repeatedly associated with higher risk of chronic pain in general populations (though older-adult specific data are fewer) [95]. In an older men’s back-pain cohort, each additional alcoholic drink per week increased odds of persistent pain (odds ratio of 1.10) [57].

Patients who are heavy smokers report higher pain intensity scores than non-smokers and report a higher number of painful sites. Smoking is involved in the aetiology of several conditions that cause chronic pain, and the relationship between smoking and chronic pain appears to be dose-related [32,97,98]. Heavy smokers tend to report more pain locations and also increased pain intensity compared with those who have never smoked. However, the evidence for a direct causal relationship between smoking and pain is not fully established. Although some have postulated that the aversive physiological effects of smoking cause or aggravate painful conditions, this has not been proven [61,95,97].

### 3.2. Environmental Factors

Environmental exposures may become particularly relevant in older adults because ageing is accompanied by greater physiological vulnerability, multimorbidity, functional limitations, and reduced capacity to adapt to adverse environmental conditions. The impact of environmental factors on chronic pain may therefore depend not only on the intensity and duration of exposure, but also on age-related susceptibility, pre-existing pain, frailty, and comorbidity. Accordingly, environmental determinants should be considered within the context of the specific vulnerabilities of older adults rather than as independent risk factors applicable uniformly across the adult population.

#### 3.2.1. Sunshine Exposure

There is some evidence to suggest a relationship between lack of sunshine, lower temperatures and pain reporting. However, pain was not deemed an inevitable consequence of such (colder) climatic conditions. Instead, the relationship may, at least in part, be mediated through lifestyle factors associated with cooler and duller days (less exercise, poorer sleep, higher reported boredom). This issue is related to some of the individual factors referred to above, namely vitamin D. A seasonal effect could suggest a role for vitamin D, low levels of which in some (but not all) studies have been shown to be related to the report of pain [61,99,100]. Furthermore, older adults may be particularly vulnerable to extreme temperatures because of impaired thermoregulation, multimorbidity, frailty, and reduced mobility, which may amplify the effects of environmental stressors on pain and functional status.

#### 3.2.2. Nature Exposure

Recent evidence indicates that nature exposure induces analgesic effects by acting on nociception-related neural processing [101]. By using functional magnetic resonance imaging (fMRI) and multi-voxel pattern analysis, it was shown that exposure to nature reduces the nociceptive activation of the thalamus, secondary somatosensory cortex, and posterior insula, indicating that the descending pain modulatory system (DPMS) may be affected. The mechanisms remain to be ascertained, but this may be important for pain managing in older adults with limited mobility or in long-term care facilities, nature also provides analgesic effects [101]. The implications of these data need to be considered in systematic reviews and meta-analysis but the high heterogeneity and risk of bias of the available studies [102] impair a formal “prescription” of nature exposure as a promising complementary pain management strategy.

### 3.3. Social Factors

Social determinants of chronic pain may have relevance in older adults because later life is accompanied by major changes in social roles, employment, household composition, and sources of social support. Compared with younger populations, retirement, widowhood, living alone, and reduced social participation may become more prominent determinants of socio-economic circumstances and social connectedness.

#### 3.3.1. Socio-Economic Background

Population studies reliably show that the prevalence of chronic pain is inversely related to socio-economic factors. Those who are socio-economically deprived are not only more likely to experience chronic pain than people from more affluent areas, but they are also more likely to experience more severe pain and a greater level of pain-related disability. People who have low levels of education, perceived income inequalities, and high levels of neighbourhood deprivation are more likely to experience chronic pain than those who have higher levels of education, less perceived income inequality, and who live in more affluent neighbourhoods. Although an individual’s socio-economic and educational background are non-modifiable, it is clear that political attention to these factors can have a great influence on the future prevalence and severity of chronic pain at a societal level [32,103,104].

Both physical and social disorders in impoverished neighbourhoods contribute to the burden of stress experiences through an overall decrease in feelings of safety among residents of the urban environment. Increasing stressors from psychosocial and environmental influences have been reported to lead to a hypersensitive stress response over time, with the potential to adversely induce pain. Adverse neighbourhood influences lead to both physical and social isolation, as well as increased stress, all of which have been found to negatively affect the chronic pain experience. In the case of chronic pain, housing status and income have been reported to be risk factors for pain because of the stress burden of homelessness or living in low-income neighbourhoods. This implies that addressing a patient’s housing status and overall state of poverty may be an important part of preventing or addressing a patient’s chronic pain because the limitations in health care access, decreased physical activity resulting from neighbourhood safety, limited access to healthy food choices, and social isolation potentially contribute to persistent chronic pain [104].

In older adults, the potential effects of urban or rural environments may be mediated by mobility, access to healthcare and social resources, transportation, and opportunities for physical activity, rather than by geographic setting alone.

#### 3.3.2. Employment, Retirement and Occupational Factors

People who are not in employment because of ill health or disability are more likely to have chronic pain than those who are employed. Occupational risk factors for chronic pain include poor job control, expectations of return to work (including fear around a recurring injury), lack of work autonomy or the ability to modify work, job satisfaction, and higher perceived level of difficulty of job requirements. Non-manual workers were less likely to report chronic pain than people who hold manual occupations. A recent study also demonstrated that chronic pain relates to working status: chronic pain was present in 79% of those who were unemployed, but only in 40% of those in paid employment and 42% of those in voluntary or unpaid work. This relationship, however, may be bidirectional in that people with chronic pain may be less likely to be in work because of their pain [32,105]. The influence of poor job control, expectations for return to work and fear of re-injury all contribute to occupational risk factors for the onset or persistence of pain. This is an important area for further study as these factors are potentially modifiable [61].

It should be noted that frequent musculoskeletal pain may increase the risk of earlier work exit and earlier retirement, with impacts on mental health, namely in the appearance of depression [106]. This recent study supports the data published in 2012 in a prospective cohort study performed in Finland, which also outlines the clear associations of disability retirement due to musculoskeletal diseases, some of them with work/occupational causes [107]. Collectively, the results show that surveillance of work and occupational factors is relevant at all ages.

Although occupational exposures are less relevant after retirement, their cumulative effects may persist into later life, particularly among individuals with previous physically demanding or hazardous occupations. Retirement represents an important life-course transition in older adults and may influence chronic pain through changes in income, daily physical activity, social participation, and access to healthcare.

#### 3.3.3. Social Isolation and Loneliness

Greater social support and social inclusion are associated with a lower risk and reduced severity of chronic pain in older adults, while social isolation and loneliness are associated with an increased chronic pain risk and pain-related disability. Social support exerts both direct and indirect effects on pain intensity, with higher levels of support linked to lower pain via improved positive affect and reduced pain catastrophising [31,108]. Social isolation and loneliness each independently increase the risk of developing chronic pain over time, with loneliness showing a particularly strong association [6,31,109]. Social isolation also predicts greater pain interference and disability, and the combination of chronic pain and social isolation has an additive effect on the risk of subsequent disability in older adults [108]. Social inclusion, including participation in social and cultural activities, is protective: high social activity and cultural engagement reduce the risk of pain and disability among older adults with chronic pain. Satisfaction with social participation and perceived social ability are negatively associated with pain interference and depressive symptoms in chronic pain populations.

In a loneliness and/or social-isolation cohort, it was found that older adults with loneliness had an odds ratio of 1.61 for developing chronic pain over seven years [31]. Social disconnectedness may amplify pain perception, reduce activity levels, impair sleep, increase depression/anxiety, and thereby serve as an indirect risk/target [110,111].

Among older adults, marital status and living arrangements may be particularly relevant because widowhood, living alone, and reduced family or community contact can increase the risk of loneliness and reduce available social support.

**Table 1:** Summary of risk factors associated with chronic pain in older adults. No formal hierarchy of evidence or certainty grading was applied.

Risk Factor Class	Risk Factor Group	References	Main Findings
**Individual** **factors**	Physical activity and sedentarism	[55,56,57,58,59,60,61]	Lifestyle behaviours can be interpreted as a risk factor for pain. Increased frequency, duration, and intensity of exercise are associated with less chronic pain in the older population.
	Body weight and nutrition	[6,12,32,61,62,63,64,65,66,67,68,69,70,71,72,73]	Unhealthy body weight is associated with increased chronic pain in older adults. There is a possible association between nutrition and chronic pain in the elderly, but it is still not established.
	Mental health and mental attitudes	[75,76,77,78,79,81]	Older adults with conditions such as depression and anxiety are at higher risk of chronic pain. Expectancy, negative affect and fear avoidance beliefs are interrelated constructs that are risk factors for future pain and disability.
	Quality of sleep	[32,61,75,82,83,84]	Poor sleep and insomnia are independent risk factors of incident pain and of worse pain outcomes. The association between poor sleep and chronic pain is bidirectional.
	Previous surgical and postoperative pain	[32,61,87,88,89,90,91,92]	Individuals with greater severity of pain at baseline were more likely to develop chronic pain and less likely to recover from it. Chronic widespread pain was associated with the number of painful regions at baseline. Postoperative chronic pain is a significant complication of many surgical procedures. Chronological age does not appear to consistently increase the risk of chronic post-surgical pain.
	Comorbidity, multimorbidity and frailty	[6,12,24,32,61,93,94,112,113,114]	There is a significant association between accumulated comorbid load and chronic pain in elderly patients. Frailty significantly impacts the risk of pain, the degree of pain, and the areas of pain in the older population.
	Smoking and alcohol use	[32,57,61,95,96,97,98]	In older men, alcohol increased persistent pain odds. Former and current heavy smokers are more likely to report more pain locations and more intense pain than never smokers.
**Environmental factors**	Sunshine exposure	[61,99,100]	Environmental factors such as reduced sunshine exposure and lower temperatures have been associated with increased pain reporting, although these conditions do not inevitably lead to pain.
	Nature exposure	[101,102]	There is a significant small-to-moderate reduction in pain associated with nature exposure, but studies exhibited moderate-to-high risk of bias and substantial heterogeneity.
**Social factors**	Socio-economic background	[32,103,104]	Among adults with any chronic pain, individuals in the lowest wealth quartile reported more pain-related disability across activity domains. Prevalence differences according to education were not significant. The onset of disabling pain in older people is influenced by local area deprivation status and perceived income inequalities.
	Employment, retirement and occupational factors	[32,61,105,107]	Occupational risk factors for chronic pain include poor job control, expectations of return to work, lack of work autonomy, job satisfaction and higher perceived level of difficulty of job requirements. Individuals who work in a lower occupational class appear to be at excess risk for chronic pain.
	Social isolation and loneliness	[31,108,109,110,111]	Social isolation and loneliness in older adults were associated with an elevated risk of chronic pain over seven years.

## 4. Age-Related Pain Sensitisation: The Rationale for Early Treatment

The risk factors discussed above may contribute to chronic pain not only by increasing peripheral pain burden, but also by promoting maladaptive changes in pain processing. Individual, environmental, and social determinants may interact through persistent nociceptive or inflammatory input and impaired pain modulation, increasing susceptibility to peripheral and central sensitisation. This provides a mechanistic link between the risk factors described in Section 3 and the rationale for early treatment discussed below.

Persistent nociceptive input or untreated neuropathic pain can lead to peripheral sensitisation, namely increased responsiveness of nociceptors. This is usually followed by central sensitisation, including hyperexcitability of spinal dorsal horn neurons, and changes in the DPMS with decreased descending inhibition and increased facilitation. Glial activation, both at the spinal dorsal horn and at brain areas of the DPMS, may occur. Collectively, this contributes to maladaptive neuroplastic changes (e.g., wind-up, temporal summation) [1,115,116,117]. In older adults, this process may be influenced by age-related changes in pain processing, including alterations in peripheral nociceptor function, central excitability, endogenous descending pain modulation, and neuroimmune signalling. These changes do not necessarily represent a uniform increase in pain sensitivity with age, but rather a reduced capacity to adapt appropriately to persistent nociceptive input. Biological ageing represents an important individual-level determinant that may integrate the effects of multiple behavioural, environmental, and social exposures on chronic pain vulnerability. Unlike chronological age, biological age reflects interindividual differences in the rate at which physiological systems deteriorate and can be estimated using biomarkers such as DNA methylation-based epigenetic clocks. Emerging evidence suggests that accelerated epigenetic ageing is associated with chronic pain in middle-aged and older adults. In community-dwelling older adults, greater epigenetic age acceleration was associated with the presence of chronic pain, greater pain during daily activities, and a higher number of painful anatomical sites [118]. Similarly, studies of individuals with knee pain and population-based cohorts have reported associations between high-impact pain and accelerated ageing measured by several generations of epigenetic clocks [119,120]. These findings raise the possibility that biological ageing may represent a mechanistic link between ageing-related vulnerability and the development or persistence of chronic pain, although the directionality and causality of this relationship remain uncertain. Epigenetic ageing is potentially influenced by modifiable exposures. DNA methylation, histone modifications, chromatin organisation, and other epigenetic processes can respond to environmental and lifestyle factors, providing a potential molecular interface between behavioural and social determinants and biological ageing. For example, insufficient sleep and insomnia have been associated with accelerated epigenetic ageing in older adults, while physical activity, correct dietary patterns, low adiposity, and other lifestyle factors may influence epigenetic age trajectories [121,122,123]. Importantly, current evidence suggests that some components of epigenetic ageing may be modifiable, although whether interventions that alter epigenetic age translate into reduced chronic pain incidence or improved pain outcomes remains unknown. A pilot randomised trial found a reduction in DNA methylation-based age following a multidomain lifestyle intervention including diet, sleep, exercise, and stress management; however, larger and longer-term studies are required before epigenetic age reversal can be considered an established clinical endpoint [124]. Thus, biological and epigenetic ageing may be viewed as a cross-cutting pathway through which individual, environmental, and social determinants converge to influence vulnerability to chronic pain.

Once central sensitisation is established, pain may persist even after resolution of the initial peripheral driver [115,116,117]. Most of the referred studies are based on animal models, but there is also evidence in humans that age-related alterations in pain modulation (reduced inhibitory control/increased excitatory actions) may predispose to faster or deeper sensitisation [125,126]. This altered pain modulation may contribute to increased vulnerability to persistent pain in some older adults [127,128]. Ageing-related changes in neuroinflammation and glial function may further contribute to maladaptive pain plasticity, while multimorbidity, frailty, sleep disturbance, and previous pain exposure may provide additional sources of nociceptive or inflammatory stress [127,128]. Together, these factors may increase vulnerability to persistent pain and reduce resilience following acute injury or disease. This provides a rationale for identifying and treating pain-related risk factors early, before maladaptive sensitisation becomes established.

Studies in humans include behavioural findings, namely using the Conditioned Pain Modulation (CPM) paradigm, which tests the ability of a pain stimulus to reduce the intensity of an initial pain (often known as “pain inhibits pain”). Research shows a significant age-dependent decline in CPM. While young adults show strong inhibitory responses, many middle-aged and older adults show little to no inhibition [128,129]. In animal models, CPM is known to be due to changes in the structure and function of DPMS [130,131]. Neuroimaging studies in humans show that ageing affects the key structures of the descending pathways to the spinal cord, namely the PAG and the rostral ventromedial medulla (RVM). Ageing is associated with a grey matter reduction, with significant volume loss occurring in the PAG but also in other areas of pain processing such as the prefrontal cortex (PFC) in older adults with chronic pain [127]. Neurochemical changes have also been proposed in the main neurotransmitters used by the DPMS, namely serotonin (5-HT) and noradrenaline (NA), which play a key role in inhibiting the transmission of nociceptive input from the spinal cord to the brain [132]. There is a progressive loss of 5-HT and noradrenergic neurons in the pathways that project to the spinal dorsal horn [127]. The opioidergic system may also be altered with accumulation of age-related pathology (like amyloid beta (Aβ) or tau proteins), which can impair the opioid-mediated modulation from the PAG, making both endogenous and pharmacological pain relief less effective [127]. Finally, there is evidence of altered connectivity in the brain of older adults, with decreased functional connectivity between areas of the DPMS (e.g., amygdala, insula, anterior cingulate cortex) and increased connectivity in sensory-related regions [133].

Overall, these findings suggest age-related changes in the balance between facilitatory and inhibitory pain processing, although the direction and magnitude of these changes vary across individuals and experimental paradigms. According to the abovementioned overall findings, early treatment of acute pain and timely recognition/treatment of neuropathic pain may reduce the risk of chronic pain development, although direct preventive evidence in older adults remains limited [134,135].

These mechanisms provide biological plausibility for the persistence and amplification of pain with ageing, but mechanistic evidence should not be interpreted as evidence that modifying these pathways prevents chronic pain in older adults.

## 5. Risk Factors for the Development of Chronic Pain in Older Adults: Implications for Prevention

Prevention of chronic pain in older adults requires complementary approaches at both the population and individual levels. At the population level, public health strategies should promote healthy ageing and reduce modifiable risk factors, whereas clinical practice should focus on identifying individual vulnerabilities and tailoring interventions accordingly [135,136]. Evidence from younger or general adult populations should not automatically be extrapolated to older adults, particularly given the potential influence of frailty, multimorbidity, polypharmacy, cognitive impairment, and functional limitations on both risk and treatment response.

Although individual risk factors may help identify older adults at increased risk of chronic pain, these associations should not be interpreted as validated prediction models. Formal prognostic models require evaluation of discrimination, calibration, clinical usefulness, and external validation [137,138], which were beyond the scope of this narrative review. Accordingly, the factors summarised in this review should be regarded as potential targets for risk stratification and prevention rather than as components of a validated predictive algorithm.

After analysing the 12 risk factors referred to above (summarised in Figure 1 and Table 1), we conclude that the scientific evidence for pain prevention in older adults needs to be further evaluated, inasmuch as specific randomised clinical trials (RCTs) evaluating the prevention of chronic pain in older adults remain limited [139]. Nevertheless, there are some studies that provide evidence, namely by identifying isolated and co-existing risk factors. Regarding isolated risk factors, physical activity is one of the main factors to consider. Meta-analyses in general populations show exercise reduces incidence of low back pain and improves outcomes in musculoskeletal pain [139,140].

For older adults, the principle applies and observational evidence suggests potential benefits of addressing these factors, although intervention evidence remains less consistent [24,75,139]. Furthermore, the guidelines (e.g., National Institute for Health and Care Excellence for neuropathic pain [134]) clearly emphasise the importance of early diagnosis and targeted therapies to avoid pain, which is important since previous pain is a risk to develop future pain. Valuable pain management strategies need to be considered, namely early interventions in primary care settings [9,35]. The logic is that early therapy reduces ongoing nociceptive and neuropathic drive and thus may prevent central sensitisation. Furthermore, in older adults, other interventions besides the pharmacological need to be considered, namely early physical therapy and behavioural interventions. Prompt recognition of neuropathic features in older adults (burning, tingling, allodynia, night pain) is important because neuropathic pain is often less responsive to standard analgesics and more prone to chronification [4,141,142]. Early use of topical agents (lidocaine patch, capsaicin) may reduce systemic exposure and adverse effects in older adults [36,143]. Persistent nociceptive input may contribute to maladaptive sensitisation and pain chronification, providing a biological rationale for timely intervention [85,86,116]. The interactions between previous pain and arising pain are complex. A Swedish study (with patients aged more than 65 years) found that having more than one pain location (odds ratio of 4.02) and higher pain severity (odds ratio of 1.79) are associated with pain persistence, though younger age is associated with new-onset pain [9].

Most studies evaluate the co-existence of the several risk factors referred to above. For example, a back-pain cohort of older men found BMI and alcohol use are associated with pain persistence [57]. There are multiple prospective cohorts in older adults indicating that risk-factor information (demographic, clinical, psychosocial, lifestyle) can stratify older individuals for pain risk/persistence. However, external validation in frail, multimorbid older adults is often lacking. The absolute predictive accuracy of risk models in older adults needs to be ascertained [137,144]. Age-related sensory changes, comorbidity, polypharmacy, cognitive impairment, and renal/hepatic changes require lower doses and slower titration [37,38], with a need for frequent review and preference for non-systemic therapies where possible.

While direct RCT evidence in older adults for early intervention preventing chronic pain is limited, observational and mechanistic data support the approach. Guideline-based early treatment may reduce transition to chronicity [134,135], and clinicians/practitioners may adopt a risk stratification and prevention model in older adults. In older adults with clinical features suggesting increased risk, targeted assessment of factors such as physical inactivity, comorbidity, frailty, sleep disturbance, obesity or underweight, mental-health problems, loneliness, smoking and alcohol consumption may help guide early intervention [135,136,144]. For those identified at higher risk of developing chronic pain, initiating targeted preventive actions (exercise referral, weight-management, sleep/psychological interventions, smoking/alcohol counselling) could attenuate the evolution. In parallel, monitoring for acute pain episodes, neuropathic symptoms, or new pain, and monitoring outcomes, such as mobility, pain frequency/severity, falls, function and quality of life, will allow for understanding the risk of a patient. This framework, though currently based on moderate-quality evidence, offers a pragmatic pathway to integrate prevention of chronic pain into health care.

It is important to note that the relevance and feasibility of preventive interventions may vary across the ageing spectrum. Younger-old adults may benefit from strategies targeting physical activity, weight management, sleep, and other modifiable lifestyle factors, whereas in old-old and oldest-old adults, particularly those who are frail, preservation of function, mobility, nutritional status, and independence may take priority. Furthermore, although multimodal management may be appropriate for older adults with complex or multifactorial chronic pain, it should not be considered universally necessary; in some patients, targeted interventions addressing a predominant pain generator or modifiable risk factor may be sufficient.

## 6. Practical Recommendations for Clinicians and Public-Health Policymakers

Preventive interventions should be adapted to the functional and cognitive status of the individual, particularly in the oldest-old and frail populations, according to chronological and biological age, frailty, functional and cognitive status, comorbidity, and the specific modifiable factors identified in each patient, rather than being applied uniformly across all older adults.

The recommendations presented in this review should be interpreted according to the type and strength of the available evidence. Some interventions have established benefits for general health, physical function, or established chronic pain, whereas direct evidence that they prevent the onset or progression of chronic pain in older adults remains limited.

For clarity, prevention strategies can be considered at different stages of the pain trajectory. Primary prevention refers to interventions aimed at preventing the onset of chronic pain in individuals without established chronic pain [135]. Secondary prevention refers to early identification and treatment of acute or subacute pain to reduce the risk of transition to chronicity [136]. Tertiary prevention concerns individuals with established chronic pain and aims to limit worsening, disability, functional decline, and related complications [136]. The evidence supporting these levels of prevention differs substantially, with direct evidence for primary and secondary prevention in older adults remaining limited.

As practical recommendations for both clinicians and policymakers, the current study emphasises the following topics:

1. *(Primary and Tertiary prevention)* Integrate pain screening into older adult assessments, based on a targeted risk-assessment approach: in primary care, geriatric clinics and community settings, ask about pain (duration ≥ 3 months, multiple sites, neuropathic features), activity limitation, sleep, mood, loneliness, comorbidities and lifestyle. Some challenges should be considered in the context of primary care. The Numeric Rating Scale (NRS) is a commonly used and valid self-report measure of pain intensity in older adults, although pain assessment should consider functional and cognitive status and no single instrument captures all relevant dimensions of pain [145,146,147]. For older adult patients with mild cognitive impairment, those scales may be complemented by the Pain Assessment in Advanced Dementia (PAINAD) scale and Doloplus-2, namely in severe cognitive impairment or advanced dementia [145,146,147].
2. *(Primary and Tertiary prevention)* Adopt a multimodal management plan for older adults with pain or at risk: prioritise non-pharmacological interventions (exercise, physical therapy, sleep hygiene, cognitive behavioural therapy, nutrition/weight management, social support) and manage pharmacotherapy carefully (start low, go slow, avoid high-risk combinations, review regularly).
3. *(Primary prevention)* Lifestyle and behavioural interventions as first-line prevention: encourage older adults to maintain or commence appropriate physical activity (strength, balance, aerobic), ensure adequate sleep and mental-health support, optimise nutrition and weight, reduce alcohol/tobacco, and address social isolation.
4. *(Primary and Tertiary prevention)* Comorbidity management: for older adults, management of osteoarthritis, diabetes, neuropathy, chronic obstructive pulmonary disease, sleep apnoea, and depression/anxiety is vital not only for general health but also for pain prevention.
5. *(Secondary prevention)* Early analgesic/adjunctive intervention for new pain: especially for neuropathic or multisite pain, early targeted treatment may plausibly reduce the risk of chronicity; coordinate with pain specialists when needed.
6. *(Primary prevention)* Health-system and policy actions: develop and support community-based exercise/referral programmes for older adults; invest in pain education for older-adult health providers; integrate pain prevention into ageing-population strategies; support research on older-adult specific pain prevention/management.
7. *(Primary, Secondary and Tertiary prevention)* Monitor and evaluate: collect data on older-adult pain prevalence, functional outcomes, falls and intervention uptake; implement quality-improvement programmes to optimise pain management and prevention in the older population.

Also, it is important to note that rather than recommending universal population screening for chronic pain risk factors in all older adults, a targeted risk-assessment approach may be more appropriate. Assessment could be considered in older adults with relevant symptoms, functional decline, previous persistent pain, multimorbidity, or other clinical characteristics suggesting increased risk. Such an approach may reduce unnecessary medicalisation and false-positive findings while allowing potential risk factors to be addressed when clinically relevant. Any prevention-oriented screening strategy should also consider potential harms and resource implications. Universal assessment of multiple risk factors may generate false-positive findings, increase medicalisation of normal ageing, and divert time and resources from other priorities in already constrained primary care settings. Moreover, evidence that systematic screening for these risk factors improves pain-related or functional outcomes is currently lacking. Until outcome-based evidence becomes available, risk assessment should therefore be individualised and proportionate to the clinical context rather than implemented as routine population-wide screening.

## 7. Conclusions

Chronic pain is highly prevalent in older adults and results from the interaction of multiple individual, environmental, and social factors. Several of these factors are potentially modifiable, supporting a prevention-oriented approach that emphasises early identification of individuals at increased risk and timely intervention, namely by a global geriatric assessment appointment. This prevention-oriented approach should therefore combine identification of potentially modifiable risk factors with consideration of the strength and consistency of causal and intervention evidence.

Preventive priorities may differ across the ageing spectrum. In younger-old adults, greater emphasis may be placed on modifying lifestyle and other preventable risk factors, whereas in old-old and oldest-old adults, particularly those with frailty or multimorbidity, preservation of physical function, mobility, nutritional status, and independence may take precedence. Prevention should therefore be individualised according to chronological and biological age, frailty, functional and cognitive status, comorbidity, and the specific risk factors identified in each person.

Overall, integrating risk factors with individualised and age-sensitive prevention may help reduce the development and persistence of chronic pain and its associated functional burden in ageing populations. Future research should evaluate and externally validate prognostic models incorporating these risk factors.

## Data Availability

No new data were created or analysed in this study. Data sharing is not applicable to this article.

## Supplementary Materials

The following supporting information can be downloaded at: https://www.mdpi.com/article/10.3390/life16091515/s1, Table S1: Scale for the Assessment of Narrative Review Articles (SANRA) for the eligible manuscripts in the Table 1.

## Abbreviations

The following abbreviations are used in this manuscript:

5-HT	Serotonin (5-Hydroxytryptamine)
Aβ	Amyloid Beta Protein
BA	Bile Acids
BMI	Body Mass Index
CPM	Conditioned Pain Modulation
CPSP	Chronic Post-Surgical Pain
DPMS	Descending Pain Modulatory System
fMRI	Functional Magnetic Resonance Imaging
FXR	Farnesoid X Receptor
IADL	Instrumental Activities of Daily Living
IASP	International Association for the Study of Pain
ICD-11	International Classification of Diseases, 11th Revision
NA	Noradrenaline
NGF	Nerve Growth Factor
PAG	Periaqueductal Grey
PAINAD	Pain Assessment in Advanced Dementia
PFC	Prefrontal Cortex
RCTs	Randomised Clinical Trials
RVM	Rostral Ventromedial Medulla
SANRA	Scale for the Assessment of Narrative Review Articles
USA	United States of America
