# Supplementary material for: Chronic Pain in Older Adults: Risk Factors and Prevention
Source: Life (Basel). 2026 Sep 11;16(9):1515. doi: 10.3390/life16091515 (PMC13608594; doi:10.3390/life16091515)
Supplement: Supplementary file 1 [file life-16-01515-s001.zip › life-4456871-supplementary.docx]

**Table S1.** Scale for the Assessment of Narrative Review Articles (SANRA) for the eligible manuscripts in the Table 1.

| **Reference** | **Importance for readership**  **(0-2)** | **Aims/questions**  **(0-2)** | **Literature search**  **(0-2)** | **Referencing**  **(0-2)** | **Scientific reasoning (0-2)** | **Data presentation (0-2)** | **Total score**  **(0-12)** |
| --- | --- | --- | --- | --- | --- | --- | --- |
| 6 | 2 | 1 | 0 | 2 | 2 | 2 | 9 |
| 32 | 2 | 2 | 0 | 2 | 2 | 2 | 10 |
| 61 | 2 | 2 | 0 | 2 | 2 | 2 | 10 |
| 62 | 2 | 2 | 0 | 2 | 2 | 2 | 10 |
| 67 | 2 | 2 | 1 | 2 | 2 | 2 | 11 |
| 68 | 2 | 2 | 0 | 2 | 2 | 2 | 10 |
| 70 | 2 | 1 | 0 | 2 | 1 | 2 | 8 |
| 71 | 2 | 2 | 0 | 2 | 2 | 2 | 10 |
| 82 | 2 | 2 | 0 | 2 | 2 | 2 | 10 |
| 89 | 2 | 2 | 0 | 2 | 2 | 1 | 9 |
| 95 | 2 | 2 | 0 | 2 | 2 | 2 | 10 |
| 96 | 2 | 2 | 0 | 2 | 2 | 2 | 10 |
| 100 | 2 | 1 | 0 | 2 | 2 | 1 | 8 |
| 104 | 2 | 2 | 1 | 2 | 2 | 2 | 11 |
| 105 | 2 | 2 | 1 | 2 | 2 | 2 | 11 |
